# Electrophysiological characterization of activation state-dependent Ca_v_2 channel antagonist TROX-1 in spinal nerve injured rats

**DOI:** 10.1016/j.neuroscience.2015.03.057

**Published:** 2015-06-25

**Authors:** R. Patel, K. Rutten, M. Valdor, K. Schiene, S. Wigge, S. Schunk, N. Damann, T. Christoph, A.H. Dickenson

**Affiliations:** a University College London, Gower Street, Department of Neuroscience, Physiology and Pharmacology, London WC1E 6BT, UK; b Grünenthal GmbH, Global Preclinical Research, 52078 Aachen, Germany

**Keywords:** ANOVA, analysis of variance, APs, action potentials, Ctrl, control, DMSO, dimethylsulfoxide, DRG, dorsal root ganglia, EGTA, ethylene glycol tetraacetic acid, HEPES, 4-(2-hydroxyethyl)-1-piperazineethanesulfonic acid, I, input, PD, post-discharge, RM, repeated measures, SNL, spinal nerve ligated, TROX-1, N-triazole oxindole, VGCCs, voltage-gated calcium channels, WDR, wide dynamic range, WU, wind-up, electrophysiology, dorsal horn, dorsal root ganglia, spinal nerve ligation, Ca_v_2.2, N-type calcium channel

## Abstract

Prialt, a synthetic version of Ca_v_2.2 antagonist ω-conotoxin MVIIA derived from *Conus magus*, is the first clinically approved voltage-gated calcium channel blocker for refractory chronic pain. However, due to the narrow therapeutic window and considerable side effects associated with systemic dosing, Prialt is only administered intrathecally. N-triazole oxindole (TROX-1) is a novel use-dependent and activation state-selective small-molecule inhibitor of Ca_v_2.1, 2.2 and 2.3 calcium channels designed to overcome the limitations of Prialt. We have examined the neurophysiological and behavioral effects of blocking calcium channels with TROX-1. *In vitro*, TROX-1, in contrast to state-independent antagonist Prialt, preferentially inhibits Ca_v_2.2 currents in rat dorsal root ganglia (DRG) neurons under depolarized conditions. *In vivo* electrophysiology was performed to record from deep dorsal horn lamina V/VI wide dynamic range neurons in non-sentient spinal nerve-ligated (SNL) and sham-operated rats. In SNL rats, spinal neurons exhibited reduced responses to innocuous and noxious punctate mechanical stimulation of the receptive field following subcutaneous administration of TROX-1, an effect that was absent in sham-operated animals. No effect was observed on neuronal responses evoked by dynamic brushing, heat or cold stimulation in SNL or sham rats. The wind-up response of spinal neurons following repeated electrical stimulation of the receptive field was also unaffected. Spinally applied TROX-1 dose dependently inhibited mechanically evoked neuronal responses in SNL but not sham-operated rats, consistent with behavioral observations. This study confirms the pathological state-dependent actions of TROX-1 through a likely spinal mechanism and reveals a modality selective change in calcium channel function following nerve injury.

## Highlights

• TROX-1 exhibits activation state-dependent inhibition of Ca_v_2.2 *in vitro*.
• TROX-1 selectively attenuates neuronal responses to mechanical stimulation.
• Anti-nociceptive effect of TROX-1 dependent on pathophysiological state.

## Introduction

Neuropathic pain represents a substantial clinical challenge with many patients failing to achieve adequate relief from currently available treatments (Breivik et al., 2006). Calcium channel modulators are one class of drug currently used and includes pregabalin and Ca_v_2.2 antagonist Prialt. However, the systemic side effects of the latter restrict delivery to an intrathecal route in cases where other treatments have failed.

Calcium influx through voltage-gated calcium channels (VGCCs) controls a multitude of physiological processes, including neurotransmitter/hormone release, membrane excitability, and activation of second messenger pathways, gene transcription and plasticity. Ca_v_2.1 (P/Q-type) and Ca_v_2.2 (N-type) channels are responsible for initiating rapid synaptic transmission. In the dorsal horn, Ca_v_2.1 and Ca_v_2.2 channels are present in distinct populations of neurons with the Ca_v_2.2 channel highly expressed in substance P positive pre-synaptic terminals of primary afferents projecting to the superficial laminae (Westenbroek et al., 1998). Furthermore, Ca_v_2.1 channels, and to a lesser extent Ca_v_2.2 channels, have been observed in the ventral horn on motor neurons. Ca_v_2.3 (R-type) channels appear to be predominantly expressed in the soma of dorsal horn neurons (Westenbroek et al., 1998). The Ca_v_2 family of channels have been implicated in mediating the release of several transmitters including dopamine, glutamate and substance P (Luebke et al., 1993; Turner et al., 1993; Terashima et al., 2013). *In vitro*, the release of CGRP, often co-expressed with substance P, in the dorsal horn is sensitive to inhibition by ω-conotoxin GVIA (Santicioli et al., 1992). Ca_v_2.2 calcium channels also mediate the release of noradrenaline from sympathetic neurons and are likely responsible for the potent hypotensive effect of parenteral ω-conotoxin GVIA (Clasbrummel et al., 1989; Molderings et al., 2000).

Genetic ablation reveals Ca_v_2.2 but not Ca_v_2.3 calcium channels are essential in basal nociception (Saegusa et al., 2000; Hatakeyama et al., 2001; Kim et al., 2001; Saegusa et al., 2001), however examining the role of Ca_v_2.1 channels in nociceptive pathways has been hampered by the severe neurological deficits and lethality of a global knockout (Jun et al., 1999). ω-conotoxin MVIIA and GVIA blockade of Ca_v_2.2 channels is effective both acutely and in models of neuropathy (Chaplan et al., 1994; Bowersox et al., 1996; Omote et al., 1996). Spinally delivered ω-agatoxin IVA has no effect on acute responses to mechanical and thermal stimulation (Sluka, 1997, 1998), but Ca_v_2.1 channels may play a more prominent role in the establishment of a sensitized state and minor effects on the maintenance (Chaplan et al., 1994; Sluka, 1998; Matthews and Dickenson, 2001). SNX-482, an antagonist of Ca_v_2.3 channels, exhibits complex pro-nociceptive and anti-nociceptive effects in the formalin test (Murakami et al., 2004).

The calcium channel antagonist N-triazole oxindole (TROX-1) has been shown to inhibit Ca_v_2.1, Ca_v_2.2 and Ca_v_2.3 VGCCs in a state- and use-dependent manner (Swensen et al., 2012). We have combined *in vitro* and *in vivo* electrophysiological approaches with behavioral assays to characterize and relate the cellular, neurophysiological and behavioral consequences of blocking calcium channels with TROX-1 to the effects of a tonic blocker. *In vivo* electrophysiology, in particular has been utilized to examine the back-translation of compounds such as pregabalin by recording from deep dorsal horn neurons in the spinal cord to study spinal processing of supra-threshold stimuli in uninjured and neuropathic conditions (Bee and Dickenson, 2008).

## Experimental procedures

### Animals

Male Sprague–Dawley rats (200–400 g), (from either the Biological Services Unit (UCL, London, UK) or Janvier, Le Genest St. Isle, France) were used for behavioral and *in vivo* electrophysiological experiments. Animals were group housed on a 12-h:12-h light–dark cycle; food and water were available *ad libitum.* All procedures described here were licensed by the appropriate governmental bodies, in compliance with local laws (UK Animals (Scientific Procedures) Act 1986 and the European Communities Council Directive of 24 November 1986 (86/609/EEC)), and were designed to reduce numbers and undue suffering in accordance with IASP ethics guidelines (Zimmermann, 1983).

### Spinal nerve ligation (SNL) surgery

SNL surgery was performed as described by Kim and Chung (1992). For rats intended for *in vivo* electrophysiology studies, surgery was performed under 2% v/v isofluorane anesthesia delivered in a 3:2 ratio of nitrous oxide and oxygen. For rats intended for behavioral studies, surgery was performed under pentobarbital anesthesia (Narcoren, 60 mg/kg intraperitoneally). Under aseptic conditions a paraspinal incision was made and the left tail muscle excised. Part of the L5 transverse process was removed to expose the L5 and L6 spinal nerves, which were then isolated with a glass nerve hook (Ski-Ry Ltd, London, UK) and ligated with a non-absorbable 6-0 braided silk thread proximal to the formation of the sciatic nerve. The surrounding muscle and skin was closed with absorbable 3-0 sutures. Sham surgery was performed in an identical manner omitting the ligation step.

### Behavioral testing

For the assessment of mechanical hypersensitivity, rats were placed on a metal mesh covered with a plastic dome and were allowed to habituate until exploratory behavior ceased. The threshold for mechanical hypersensitivity was determined with an electronic von Frey anesthesiometer (Somedic AB, Malmö, Sweden) using the median of five consecutive measurements (inter-measurement interval 1–2 min). Animals with ipsilateral withdrawal thresholds >30 g and/or contralateral withdrawal thresholds <50 g were excluded from the experiment as they did not develop a tactile hypersensitivity (on average 1/12 animals is excluded). Animals were tested before and 0.5, 1, and 3 h after administration of the test compounds (intrathecal Prialt (ω-conotoxin MVIIA, Bachem AG, Bubendorf, Switzerland) dissolved in 0.9% saline, 5-μl dose; intrathecal racemic TROX-1 ((3R)-5-(3-chloro-4-fluorophenyl)-3-methyl-3-(pyrimidin-5-ylmethyl)-1-(1H-1,2,4-triazol-3-yl)-1,3-dihydro-2H-indol-2-one) (synthesized in house; Grünenthal GmbH, Germany), 5-μl dose; subcutaneous TROX-1 (5 ml/kg), dissolved in 10% dimethylsulfoxide (DMSO), 5% cremophor and 5% glucose solution). Intrathecal dosing was performed as previously described (Mestre et al., 1994). Drugs or vehicle were tested 2–5 weeks after surgery (one test per week) in a counterbalanced within-group design. Animals were randomly assigned to sham or SNL groups and to treatment conditions. Behavioral testing was performed with the experimenter blinded to the treatment conditions.

### *In vitro* electrophysiology

Cryopreserved neonatal (P2-3) rat dorsal root ganglia (DRG) were obtained from Lonza Group Ltd, Cologne, Germany. After thawing a vial of cells at 37 °C the cells were mixed drop-wise with 7.75 ml of pre-warmed (37 °C) Medium A (Primary Neuron Basal Medium 200 ml, Glutamin 2 ml, Gentamycin/Amphotericin 0.2 ml, NSF-1 4 ml) over a 2-min time frame. Cells were then transferred to Poly-d-Lysin/Laminin coverslips and incubated at 37 °C, 5% CO2, 95% humidity for 4 h. Afterward Medium A was replaced with 90 % Medium B (Medium A, FUDR 5 μg/ml, UDR 5 μg/ml) and cells were incubated for 4–7 days until use. Medium B was changed to 50 % every 3 days.

Whole-cell patch-clamp experiments were carried out with a HEKA EPC 10 patch-clamp amplifier (HEKA Elektronik Dr. Schulze GmbH, Germany). Borosilicate patch electrodes with resistances from 3 to 5 MΩ were used when filled with internal solution containing (in mM): 130 CsCI, 2.7 MgCI_2_, 9 EGTA, 9 HEPES, 4 MgATP, 0.3 GTP (Tris), 14 phosphocreatine (Tris); pH 7.4 adjusted with CsOH. The extracellular solution contained (in mM) 150 TEA-Cl, 5 CaCl_2_, 0.5 MgCI_2_, 10 HEPES and 10 glucose. The pH was adjusted to 7.3 with TEA-OH.

DRG neurons were voltage clamped at a holding membrane potential of −90 mV for closed state or at −50 mV for inactivated state experiments. The voltage-clamp protocol consisted of a 100 ms pre-pulse to −50 mV to inactivate T-type channels followed by a 50 ms step to +10 mV. Sweeps were repeated once every 15 s and were run continuously to activate Ca_v_2.2 channels. All currents were leak subtracted using a standard subtraction protocol. After a stable Ca^2+^ current was obtained, DRG neurons were superfused with 100 nM isradipine, 300 nM agatoxin IVA, and 150 nM SNX-482 to minimize the contribution of other non-N-type calcium channels during the whole experiment. Compounds were directly applied to the cell by a multi-valve perfusion system (ALA Scientific Instruments, Farmingdale, NY, USA) until steady-state block was achieved. Prialt (ω-conotoxin MVIIA, Bachem AG, Bubendorf, Switzerland) and TROX-1 (Grünenthal GmbH, Germany) was first dissolved in saline (0.9% NaCl) or 100% DMSO, respectively, and then adjusted to a final concentration lower than 0.3% DMSO.

### *In vivo* electrophysiology

*In vivo* electrophysiology was conducted as previously described (Urch and Dickenson, 2003). Spinal nerve-ligated rats were used between days 15 and 18-post surgery. Animals were anesthetized with 3.5% v/v isofluorane delivered in a 3:2 ratio of nitrous oxide and oxygen. Once areflexic, a tracheotomy was performed and rats were subsequently maintained on 1.5% v/v isofluorane for the remainder of the experiment. Rats were secured in a stereotaxic frame and a laminectomy was performed to expose L4–L5 segments of the spinal cord. Extracellular recordings were made from deep dorsal horn wide dynamic range (WDR) spinal neurons (lamina V/VI) with receptive fields on the glabrous skin of the toes using parylene-coated tungsten electrodes (A-M Systems, Sequim, WA, USA). Neurons were identified as WDR by confirming responses to light brushing of the receptive field, noxious punctate mechanical stimulation and noxious thermal stimulation.

Electrical stimulation of WDR neurons was delivered transcutaneously via needles inserted into the receptive field. A train of 16 electrical stimuli (2-ms pulses, 0.5 Hz) was applied at three times the threshold current for C fiber activation. Responses evoked by Aβ- (0–20 ms), Aδ- (20–90 ms) and C-fibers (90–350 ms) were separated and quantified on the basis of latency. Neuronal responses occurring after the C-fiber latency band were classed as post-discharge (PD). The input (I) and the wind-up (WU) were calculated as Input = (action potentials evoked by first pulse) × total number of pulses (16), wind-up = (total action potentials after 16 train stimulus) − Input. The receptive field was also stimulated using a range of natural stimuli (brush, von Frey filaments – 2, 8, 15, 26 and 60 g and heat – 35, 42, 45 and 48 °C) applied over a period of 10 s per stimulus and the evoked response quantified. The heat stimulus was applied with a constant water jet onto the center of the receptive field. One hundred microliter acetone and ethyl chloride (Miller Medical Supplies, Newport, UK) were applied as an evaporative innocuous cooling and noxious cooling stimulus respectively (Leith et al., 2010). Evoked responses to room temperature water were subtracted from acetone and ethyl chloride-evoked responses to control for concomitant mechanical stimulation during application.

Data were captured and analyzed by a Cambridge Electronic Design 1401 interface coupled to a computer with Spike 2 software (Cambridge, United Kingdom) with post-stimulus time histogram and rate functions. After three consecutive stable baseline responses to natural stimuli (<10% variation, data were averaged to give control values), animals were injected subcutaneously into the contralateral flank with 20 mg/kg TROX-1. Responses to electrical and natural stimuli were measured 10, 30 and 50 min post dosing. For spinal dosing, 0.1, 1 and 10 μg of TROX-1 was cumulatively applied directly onto the cord in a volume of 50 μl. The vehicle for spinally applied drug was diluted to <1% cremophor and <1% DMSO. One neuron was characterized per rat.

### Statistics

Statistical analyses were performed using SPSS v22 (IBM, Armonk, NY, USA). Behavioral time courses, and differences in mechanical and thermal coding of neurons were tested using a 2-way repeated measures (RM) analysis of variance (ANOVA), followed by a Bonferroni post hoc test for paired comparisons. Sphericity was tested using Mauchly’s test, the Greenhouse-Geisser correction was applied if violated. Dynamic brush, cold stimulation and electrical parameters were compared with a paired Student’s *t*-test or 1-way RM ANOVA, followed by a Bonferroni post hoc test for paired comparisons.

## Results

### Intrathecal TROX-1 attenuates mechanical hypersensitivity in spinal nerve-ligated rats

**Fig. 1:**
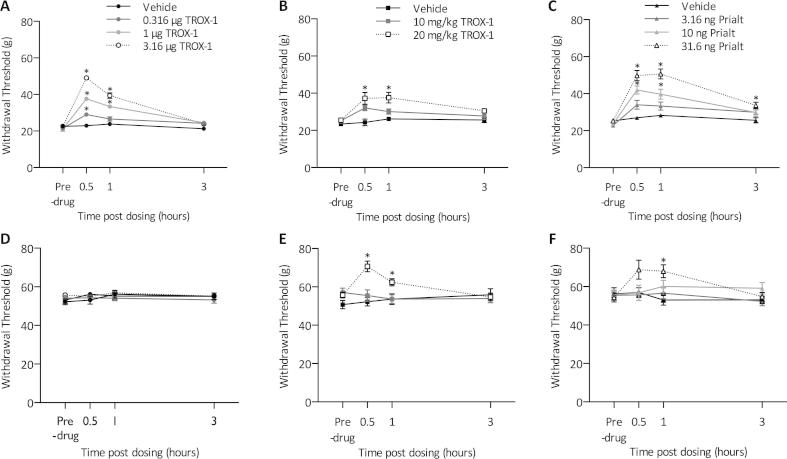
TROX-1 and Prialt increase mechanical withdrawal thresholds in spinal nerve-ligated rats. (A) Effect of intrathecal TROX-1 on ipsilateral withdrawal thresholds. (B) Effect of subcutaneous TROX-1 on ipsilateral withdrawal thresholds. (C) Effect of intrathecal Prialt on ipsilateral withdrawal thresholds. (D) Effect of intrathecal TROX-1 on contralateral withdrawal thresholds. (E) Effect of subcutaneous TROX-1 on contralateral withdrawal thresholds. (F) Effect of intrathecal Prialt on contralateral withdrawal thresholds. Asterisks denote statistically significant difference from vehicle group. Data represent mean ± SEM, *n *= 10, ^∗^*P* < 0.05.

Rats were examined at least 14 days post injury for signs of behavioral hypersensitivity following sham or SNL surgery. SNL rats displayed guarding behavior of the injured ipsilateral hind paw, a feature that was absent on the uninjured contralateral side and in sham-operated rats. SNL rats, but not sham, displayed significantly reduced withdrawal thresholds to punctate mechanical stimulation. Intrathecal TROX-1 (Fig. 1A) and subcutaneous TROX-1 (Fig. 1B) dose dependently increased mechanical withdrawal thresholds in SNL rats (2 way RM ANOVA, *P* < 0.01). Intrathecal Prialt also dose dependently increased withdrawal thresholds in SNL rats (2 way RM ANOVA, *P* < 0.01) (Fig. 1C), however, the 31.6-ng dose induced respiratory depression, flat posture, ptosis and sniffing behaviors. On the nerve-injured side, intrathecal TROX-1 displayed comparable efficacy to Prialt. In contrast, intrathecal TROX-1 had no effect on contralateral withdrawal thresholds (Fig. 1D), whereas Prialt increased withdrawal thresholds at the highest dose (Fig. 1F). Surprisingly, systemically delivered TROX-1 increased contralateral withdrawal thresholds at the highest dose tested (Fig. 1E). This could be attributed to non-spinally mediated effects of TROX-1. No obvious signs of motor deficits were apparent (Rota-rod, data not shown). Vehicle treatment had no effect on withdrawal thresholds in sham or SNL animals (data not shown).

### TROX-1 exhibits activation state-dependent block of Ca_v_2.2 calcium channels in rat dorsal root ganglion neurons *in vitro*

Electrophysiological experiments were performed to record Ca_v_2.2 calcium currents in rat DRG neurons. Ca_v_3 calcium channels were inactivated by voltage pre-pulses, and Ca_v_2.1, Ca_v_2.3 and Ca_v_1 calcium currents were blocked by a mixture of pharmacological blockers (Isradipine (100 nM), Agatoxin (300 nM) and SNX-482 (150 nM)). The remaining calcium current was anticipated to be carried by Ca_v_2.2 channels and was challenged with Prialt and TROX-1. The average current for resting state Ca_v_2.2 currents was 551.7 ± 56.5 pA and 285.6 ± 40.5 pA for the inactivated state.

**Fig. 2:**
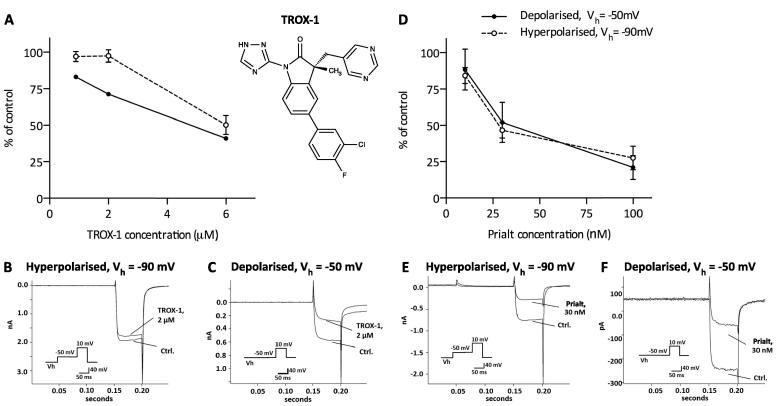
TROX-1 preferentially inhibits Ca_v_2.2 currents in rat dorsal root ganglion neurons under depolarized conditions. Experiments conducted in the presence of isradipine (100 nM), Agatoxin (300 nM) and SNX-482 (150 nM) to block non-Ca_v_2.2 channels. (A) Effect of TROX-1 on calcium currents under depolarized and hyperpolarised conditions. Representative traces of the effects of 2 μM TROX-1 on calcium currents under hyperpolarized (B) and depolarized (C) conditions. (D) Effect of Prialt on calcium currents under depolarized and hyperpolarized conditions. Representative traces of the effects of 30 nM Prialt on calcium currents under hyperpolarized (E) and depolarized (F) conditions. Data represent mean ± SEM, *n *= 4–5. (Ctrl – control).

In depolarized DRG neurons, the activation state-dependent blocker TROX-1 exhibited a concentration-dependent inhibition of Ca_v_2.2 calcium currents, whereas TROX-1 only induced a weak concentration-dependent block when channels were in a closed state (*V_h_* = −90 mV) (Fig. 2A). Under hyperpolarised conditions, 2 μM TROX-1 inhibited 2.6 ± 4.2 % (Fig. 2B) of Ca_v_2.2 currents compared to 28.6 ± 1.5 % (Fig. 2C) under depolarized conditions.

The non-state dependent blocker Prialt induced a concentration-dependent block of Ca_v_2.2 calcium channels with a near complete block at both holding potentials (Fig. 2D). At a holding potential of *V_h_* = −90 mV a residual calcium current of 46.6 ± 5.43% (54.4% inhibition) was determined with 30 nM Prialt (Fig. 2E). Under depolarized conditions 30 nM Prialt reduced the calcium current (*V_h_* = −50 mV) to 52.0 ± 13.78% (48% inhibition) (Fig. 2F).

### Systemic TROX-1 inhibits neuronal responses to mechanical stimulation in spinal nerve-ligated rats

*In vivo* electrophysiology was subsequently performed to examine the effect of TROX-1 on mechanical and thermal coding of lamina V/VI spinal neurons under uninjured and neuropathic conditions. A total of 25 neurons were characterized, Table 1 summarizes neuronal depths recorded from and evoked baseline responses. Neurons were characterized from depths relating to deep dorsal horn laminae (Watson et al., 2009) and were selected on the basis of responses to noxious heat, dynamic brushing and noxious mechanical stimulation. No significant difference was observed in the number of Aβ-, Aδ- and C- fiber evoked action potentials (APs) or the electrical thresholds for activation of A- and C- fibers (unpaired Student’s *t*-test). Thermally and mechanically evoked responses of neurons were also similar between sham and SNL rats (2 way RM ANOVA, *P *> 0.05).

**Fig. 3:**
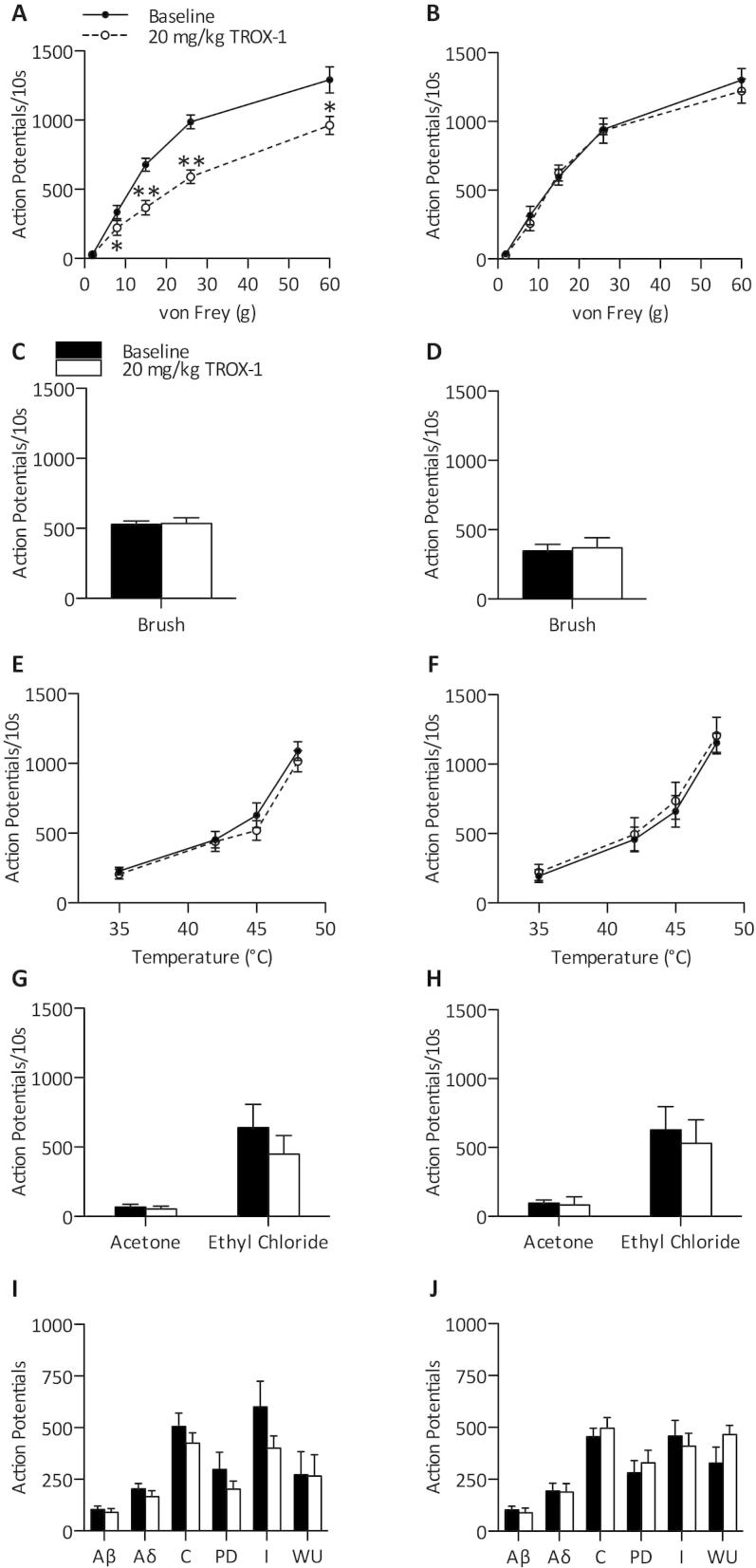
Systemic TROX-1 selectively inhibits mechanical coding of dorsal horn lamina V/VI neurons in spinal nerve-ligated rats. Following stable baseline recordings (<10% variation), SNL (*n *= 6) or sham (*n *= 5) rats were dosed subcutaneously with 20 mg/kg TROX-1. Neuronal responses to punctate mechanical (A, B), dynamic brushing (C, D), heat (E, F), innocuous and noxious cooling (G, H), and electrical stimuli (I, J) were recorded. For natural stimuli, responses were quantified over a 10-s period. Following repeated electrical stimulation, action potentials were separated according to latency: Aβ: 0–20 ms, Aδ: 20–90 ms, C: 90–350 ms, Post-discharge >350 ms. Input and wind-up calculated as described in Experimental procedures. Left panels – SNL, right panels – sham). Data displayed as maximum change from baseline. Data represent mean ± SEM. ^∗^*P* < 0.05, ^∗∗^*P* < 0.01. I – input, WU – wind-up, PD – post-discharge.

Following isolation and characterization of single WDR neurons, rats were dosed subcutaneously with 20 mg/kg TROX-1. In SNL rats, TROX-1 suppressed mechanical coding of dorsal horn neurons with significantly reduced neuronal responses to low -threshold and supra-threshold stimuli (2-Way RM ANOVA *P* < 0.001, followed by Bonferroni post hoc test) (Fig. 3A). However, no effect was observed on mechanical coding in sham-operated rats (2-Way RM ANOVA *P* > 0.05) (Fig. 3B). Neuronal responses to dynamic brushing (paired Student’s *t*-test, *P *> 0.05) (Fig. 3C, D), heat (2-Way RM ANOVA *P *> 0.05) (Fig. 3E, F) and cold stimulation (paired Student’s *t*-test, *P *> 0.05) (Fig. 3G, H) were also unaffected in both SNL and sham rats. A train of electrical stimuli was applied to examine the effect of TROX-1 on pre- and post-synaptic measures of activity. The total number of evoked APs attributed to Aβ-, Aδ- and C- fibers did not significantly differ to baseline in SNL or sham rats. Indicators of neuronal excitability, I, WU and PD were also unaffected (paired Student’s *t*-test, *P *> 0.05) (Fig. 3I, J). No effect of the high dose of TROX-1 was observed on any of the neuronal responses in uninjured sham rats despite the same dose being highly effective and selective for mechanical yet not heat-evoked firing after nerve injury.

### Spinal dosing replicates systemic effect of TROX-1 on mechanically evoked neuronal responses in spinal nerve-ligated rats

**Fig. 4:**
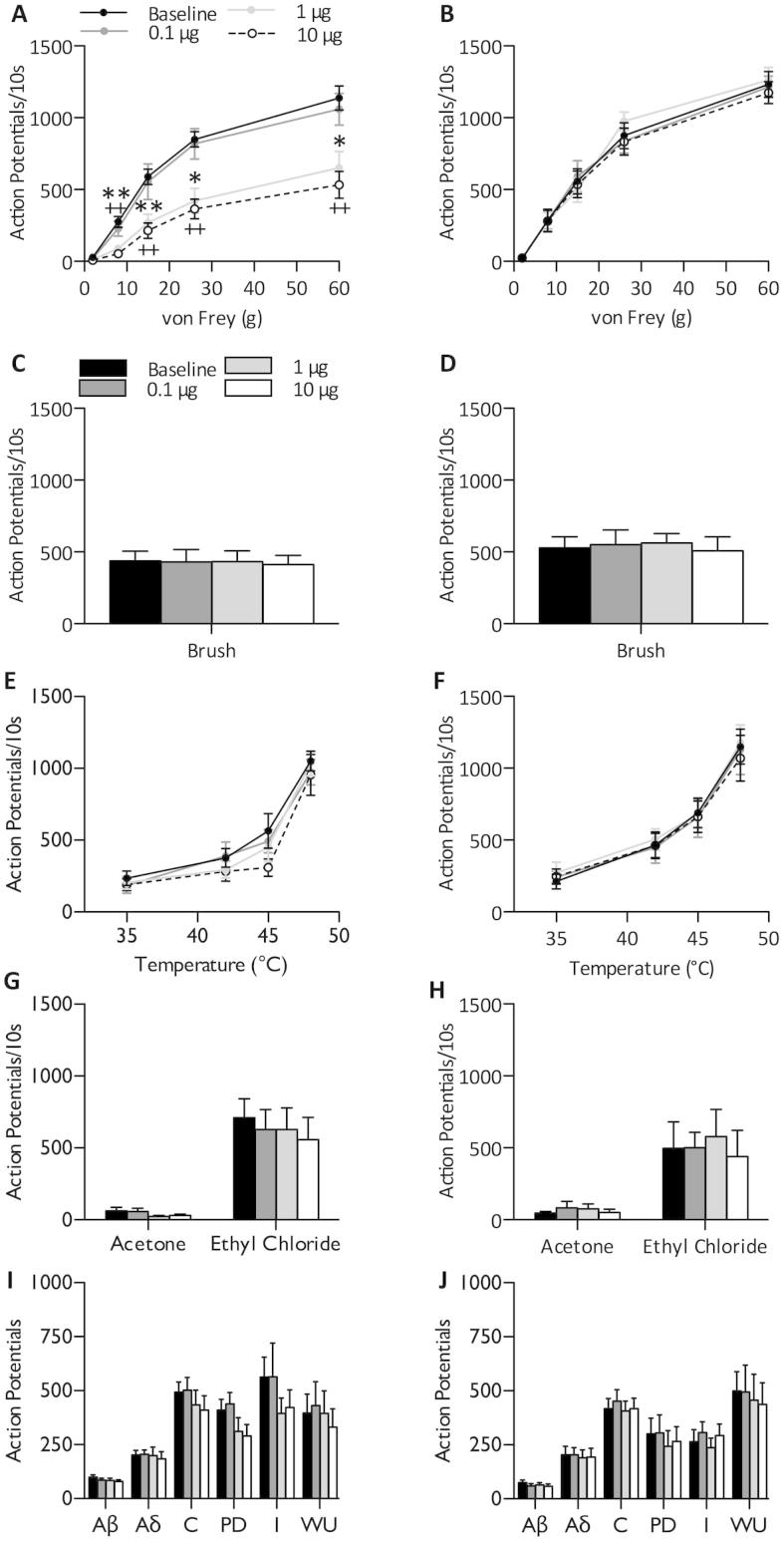
Spinal TROX-1 selectively inhibits mechanical coding of dorsal horn lamina V/VI neurons in spinal nerve-ligated rats. Following stable baseline recordings (<10% variation), SNL (*n* = 8) or sham (*n* = 6) rats were dosed spinally with 0.1, 1 and 10 μg TROX-1 in a volume of 50 μl. Neuronal responses to punctate mechanical (A, B), dynamic brushing (C, D), heat (E, F), innocuous and noxious cooling (G, H), and electrical stimuli (I, J) were recorded. For natural stimuli, responses were quantified over a 10-s period. Following repeated electrical stimulation, action potentials were separated according to latency: Aβ: 0–20 ms, Aδ: 20–90 ms, C: 90–350 ms, Post-discharge >350 ms. Input and wind-up calculated as described in Experimental procedures. Asterisks (^∗^) denote significant differences between baseline and 1 μg. (+) denotes significant differences between baseline and 10 μg. Left panels – SNL, right panels – sham). Data displayed as maximum change from baseline. Data represent mean ± SEM. ^∗^*P* < 0.05, ^∗∗^*P* < 0.01. I – input, WU – wind-up, PD – post-discharge.

The mechanism of TROX-1 was further investigated by examining the cumulative effects of 0.1, 1- and 10-μg TROX-1 applied directly onto to the spinal cord. Spinal TROX-1 dose dependently inhibited neuronal responses to punctate mechanical stimulation of the receptive field in SNL rats (2 way RM ANOVA, *P* < 0.001) (Fig. 4A) with no effect observed in sham rats (2-way RM ANOVA, *P *> 0.05) (Fig. 4B). As previously observed through a systemic route of administration, TROX-1 did not alter evoked responses to brushing (1-way RM ANOVA *P *> 0.05) (Fig. 4C, D), heat (2-way RM ANOVA *P *> 0.05) (Fig. 4E, F), cold (1-Way RM ANOVA *P *> 0.05) (Fig. 4G, H) or electrical stimulation (1-way RM ANOVA *P *> 0.05) (Fig. 4I, J) of the receptive field in either conditions.

## Discussion

This study extends previous observations of the effects of TROX-1 in nociceptive assays in providing a neuronal correlate of behavioral responses to threshold and supra-threshold stimulation in a model of neuropathic pain. Prialt, unlike TROX-1, displays no preference for activation state, or frequency-dependent inhibition (Feng et al., 2003). *In vivo*, TROX-1 selectively attenuates mechanical hypersensitivity only after neuropathic injury, unlike calcium channel toxins.

We have previously examined the effects of antagonists of the Ca_v_2.2 (ω-conotoxin GVIA), Ca_v_2.1 (ω-agatoxin IVA) and Ca_v_2.3 (SNX-482) channels on spinal neuronal activity in nerve-injured and uninjured conditions. All three antagonists inhibited neuronal responses to mechanical, heat and electrical stimuli, including wind-up, in SNL rats to various degrees, though SNX-482 had minimal effects in sham rats (Matthews and Dickenson, 2001; Matthews et al., 2007). The increased potency of ω-conotoxin GVIA and SNX-482, but not ω-agatoxin IVA, in SNL rats identifies significant neuroplastic changes that modulate Ca_v_2.2 and Ca_v_2.3 function and/or expression after injury (Matthews and Dickenson, 2001; Matthews et al., 2007). The anti-hyperalgesic effect of TROX-1 is absent in Ca_v_2.2 null mice demonstrating a predominant role of this particular channel in inflammatory heat hyperalgesia (Abbadie et al., 2010).

We have recorded from WDR lamina V/VI neurons; the firing patterns of these neurons have been shown to correlate with intensity and temporal aspects of pain in animals and human subjects (Coghill et al., 1993; Sikandar et al., 2013). TROX-1 exhibits pathophysiological state-dependent activity and selectively suppresses mechanical coding of WDR neurons in SNL rats. *In vitro*, TROX-1 increasingly inhibits calcium currents following repeated trains of depolarizations thereby demonstrating use-dependency (Swensen et al., 2012). In contrast, TROX-1 had no impact on wind-up potentiation of dorsal horn neurons following repeated electrical stimulation *in vivo*. The nature of this stimulus is more akin to supra-threshold stimulation and involves synchronized activation of primary afferent terminals in the receptive field. The discord between the *in vitro* and *in vivo* scenarios could be explained by the differing stimulus parameters (*in vitro* − 20 × 25 ms pulse, 2 Hz. *In vivo* – 16 × 2 ms pulse, 0.5 Hz). Wind-up occurs optimally between 0.5 and 2 Hz (Herrero et al., 2000). In this respect, the stimulus frequencies were relatively comparable between the two studies. Additionally, the proportion of channels entering an inactivated state increases with prolonged stimulus duration (Freeze et al., 2006). Mechanical stimuli applied for 10 s results in prolonged depolarizations at threshold levels of intensity and thus, natural stimulation may be more amenable to inhibition in comparison to a rapid electrical stimulus *in vivo*. Also, an important caveat to note is that expression systems do not fully recreate regulation of channel function.

Dynamic mechanical and cold allodynia are frequent features of several neuropathies (Maier et al., 2010), however brushing- and cooling-evoked neuronal responses were not inhibited by TROX-1. Surprisingly, heat-evoked responses were also unaffected by TROX-1 given the anti-nociceptive effects of TROX-1 in inflammation-induced heat hyperalgesia (Abbadie et al., 2010). This perhaps reflects divergent mechanisms between inflammatory hyperalgesia and nerve injury models. A differential regulation of pre-synaptic Ca_v_2.2 function may underlie the modality-selective actions of TROX-1 in SNL rats.

Functional diversity of Ca_v_2.2 calcium channels is achieved through alternate splicing of the C-terminus. In particular the e37a and e37b variants are highly expressed in nociceptors and whereas the former is associated with mechanical and thermal hypersensitivity, the latter only influences mechanical hypersensitivity (Altier et al., 2007). The e37a variant, compared to e37b, exhibits increased channel open time and inactivates more slowly (Castiglioni et al., 2006). Channels exhibiting these biophysical properties may be more favorable for inhibition by a state-dependent antagonist. However, many thousands of splice variants exist, which may be differentially expressed after injury, and could have modality-selective consequences for sensory transduction. At the mRNA level, Ca_v_2.2 channels do not appear up-regulated in DRG neurons following nerve ligation while some splice variants are down-regulated (Altier et al., 2007). In contrast, an increase in the immunoreactivity of the pore forming α_1B_ subunit in the superficial laminae has been reported (Cizkova et al., 2002). This is likely due to the up-regulation of the α_2_δ-1 subunit that occurs after nerve injury (Luo et al., 2001), which is also the molecular target for the anti-hyperalgesic effects of the gabapentinoids (Field et al., 2006; Patel et al., 2013). Elevated levels of the α_2_δ-1 subunit in the central terminals of primary afferent fibers enhances trafficking of channels to synapses in addition to determining kinetics of transmitter release (Hoppa et al., 2012). Consistent with this role, synaptosomal levels of Ca_v_2.2 are reduced in α_2_δ-1 knockout mice (Patel et al., 2013). Although predominantly expressed in unmyelinated DRG neurons under normal conditions, the α_2_δ-1 subunit is up-regulated in neurons of all types after injury (Bauer et al., 2009). The increase in trafficking and inactivation rate of calcium channels by α_2_δ-1 likely enhances pre-synaptic terminal excitability and facilitates excitatory transmission by supporting rapid transition between active and inactive states (Li et al., 2006). Differential expression of α_2_δ-1 splice variants also occurs following SNL, though these variants exert similar effects on the biophysical properties of Ca_v_2 currents (Lana et al., 2013). Although not consistently observed, expression of the β3 subunit may also increase after nerve injury (Li et al., 2012; Patel et al., 2013), which could further influence the rate of channel activation and inactivation (Castellano et al., 1993), resulting in enhanced calcium currents in small diameter DRG neurons (Li et al., 2012). Overall, the magnitudes of effect for systemic pregabalin and TROX-1, at similar doses, on lamina V/VI neuronal responses are similar, although the former additionally inhibited thermal responses (Bee and Dickenson, 2008).

In addition, descending facilitatory and inhibitory monoaminergic pathways converging onto the dorsal horn may also influence the excitability of pre-synaptic terminals. Descending serotonergic inhibitory pathways terminating on metabatropic 5-HTRs can directly, or indirectly through inhibitory interneurons, exert inhibitory influences on pre-synaptic calcium channel function and subsequent transmitter release (Brenchat et al., 2010; Choi et al., 2012). G-protein-coupled receptor moderation of primary afferent terminal excitability could additionally occur through regulation of potassium currents consequently hyperpolarizing pre-synaptic terminals (Yagi and Sumino, 1998). Numerous studies have identified deficits in inhibitory drive following a neuropathic injury (Moore et al., 2002; Rahman et al., 2008; Hughes et al., 2013). Additionally, descending facilitatory pathways activating ionotropic 5-HT_3_Rs will have further depolarizing effects on pre-synaptic terminals. Spinally delivered ondansetron, a 5-HT_3_R antagonist, inhibits mechanically evoked responses with minimal effects on heat-evoked responses in SNL rats (Suzuki et al., 2004). Spinalization of SNL rats ablates mechanical hypersensitivity but not heat hypersensitivity indicating the latter is likely dependent on spinal disinhibition whereas the former requires intact spinal-supraspinal circuits (Ossipov et al., 2000). One possibility is that a combination of neuroplastic changes in primary afferent excitability coupled with alterations in activity in descending pathways underlies the pathophysiological and modality-selective actions of TROX-1 in SNL rats. This would be consistent with efficacy in the sodium iodoacetate-induced osteoarthritis model (Abbadie et al., 2010), which can be characterized by a neuropathic component resulting in hypersensitivity in areas of the dermatome outside the primary area of injury (Combe et al., 2004). A time-dependent loss of noradrenergic inhibition and an increased descending facilitatory drive has been demonstrated in this model (Rahman et al., 2009; Burnham and Dickenson, 2013).

Our data suggest spinally expressed calcium channels are critical for the anti-hyperalgesic effects of TROX-1. Ca_v_2.2 calcium channels at the site of injury may contribute to afferent dysfunction, generation of ectopic discharges and hyperexcitability (Xiao and Bennett, 1995), though this does not appear consistent across models (Chaplan et al., 1994). Injection of ω-conotoxin GVIA into the central nucleus of the amygdala is pro-nociceptive in the early phase of the formalin test with no effect on the secondary phase supporting a role for the central nucleus in tonically inhibiting acute nociceptive drive (Finn et al., 2003). Intra-rostral ventromedial medulla injections of ω-conotoxin MVIIA, but not ω-agatoxin IVA, alleviates mechanical hypersensitivity following SNL implicating Ca_v_2.2 over Ca_v_2.1 channels in modulating activity in descending pathways (Urban et al., 2005). However, the precise mechanism at this site remains unclear. These findings do not entirely preclude non-spinally mediated effects of TROX-1.

Several clinical trials have been undertaken to examine efficacy of state-dependent calcium channel antagonists in chronic pain patients (Clinical Trials Identifiers: NCT01848730, NCT01655849). Compounds such as TROX-1 have substantial advantages over peptide antagonists by being orally bioavailable and blood–brain barrier penetrant. State-dependent channel antagonists are a promising area for selectively inhibiting aberrant neuronal activity in neuropathic pain. From a translational perspective, further pre-clinical characterizations of the effects on nociceptive processing may help shape future trials and act as better predictors for success.

## Funding

This study was funded by Grünenthal. RP is supported by the Biotechnology and Biological Sciences Research Council [BB/H016597/1].

## Author Contributions

All authors have read and approved this manuscript. R.P., K.R., S.S., N.D. and A.H.D. conceived and planned experiments. R.P., T.C., K.S., S.W. and M.V. participated in the acquisition of data. R.P., K.S., K.R., M.V. performed data analysis. A.H.D. and S.S. contributed reagents/analytical tools. R.P., A.H.D., K.R., M.V., N.D., and S.S. wrote the manuscript.

## Conflicts of interest

A.H.D. has received research funding and speaker fees from Grünenthal.

## Figures and Tables

**Table 1:** Baseline characterizations of deep dorsal horn wide dynamic range (WDR) neurons from sham and SNL rats. Range of neuronal depths recorded from in parentheses. Data represent mean ± SEM. (APs – action potentials)

	Sham *n *= 11	SNL *n* = 14
Depth (μm)	773 ± 32.88	616 ± 35.32
	(670–910)	(490–880)
A threshold (mA)	0.03 ± 0.01	0.06 ± 0.01
C threshold (mA)	0.63 ± 0.17	0.47 ± 0.13
Aβ-evoked (APs)	87 ± 10.97	101 ± 8.94
Aδ-evoked (APs)	199 ± 25.61	203 ± 15.82
C-evoked (APs)	435 ± 30.65	499 ± 37.25
Post-discharge (APs)	292 ± 45.64	362 ± 46.24

Brush (APs)	445 ± 53.61	477 ± 40.33
2 g (APs)	28 ± 4.31	29 ± 4.77
8 g (APs)	299 ± 49.50	302 ± 29.70
15 g (APs)	575 ± 52.39	627 ± 37.02
26 g (APs)	906 ± 50.63	909 ± 40.27
60 g (APs)	1264 ± 59.62	1203 ± 64.02

35 °C (APs)	203 ± 32.84	232 ± 28.63
42 °C (APs)	461 ± 61.18	412 ± 43.97
45 °C (APs)	676 ± 71.89	595 ± 74.33
48 °C (APs)	1151 ± 70.01	1068 ± 46.29

Acetone (APs)	74 ± 16.26	65 ± 15.71
Ethyl chloride (APs)	570 ± 118.25	680 ± 100.73
